# Left-right commissural aortic root enlargement facilitates valve-in-valve transcatheter aortic valve implantation: A computed tomographic analysis

**DOI:** 10.1016/j.xjtc.2025.10.014

**Published:** 2025-10-30

**Authors:** Jahangir H. Charania, Logan Atkinson, Sorush Rokui, Daniel R. Wong

**Affiliations:** a Division of Cardiology, Royal Columbian Hospital, New Westminster, British Columbia, Canada; b Division of Cardiac Surgery, University of British Columbia, Vancouver, British Columbia, Canada; c Division of Cardiac Surgery, Royal Columbian Hospital, New Westminster, British Columbia, Canada

**Keywords:** aortic root enlargement, surgical aortic valve replacement, transcatheter aortic valve implantation, transcatheter aortic valve replacement, valve-in-valve

## Abstract

**Objective:**

Aortic root enlargement (ARE) at the left-right (LR) commissure may mitigate risk of coronary obstruction during future valve-in-valve (VinV) transcatheter aortic valve implantation (TAVI) by creating space for blood flow behind the LR surgical valve strut into the adjacent sinuses. We analyzed computed tomography (CT) scans after LR ARE to determine theoretical candidacy for future VinV TAVI.

**Methods:**

All patients undergoing LR ARE and bioprosthetic surgical aortic valve replacement (SAVR) between February 2023 and November 2024 were reviewed retrospectively. Postoperative CT scans were analyzed, modeling for both balloon-expandable (BEV) and self-expanding (SEV) valves. Risk of coronary obstruction was based on virtual transcatheter valve to coronary (VTC) and sinotubular junction (VTSTJ) distances, and measurements of the LR ARE patch.

**Results:**

There were 32 patients (62% female, 44% inpatient) who had LR ARE with bioprosthetic SAVR, including 22% with a concomitant Y ARE. There was no 30-day mortality or stroke. Postoperative mean gradient was 5.5 mm Hg. Overall, 85% of valves were 23 or 25 mm, representing upsizing by 1.7 ± 0.7 sizes. The patch was 26.2 ± 4.7 mm tall, with 6.2 ± 2.4 mm of space behind the LR strut. If a SEV were used, 94% would be safe for VinV TAVI with low risk of coronary obstruction; if balloon inflation were needed during TAVI (such as for BEV), 77% could have safe VinV TAVI.

**Conclusions:**

In this CT analysis, LR ARE carried low risk of coronary obstruction for future VinV TAVI and may benefit patients undergoing bioprosthetic SAVR.

## Graphical Abstract

**Figure:**
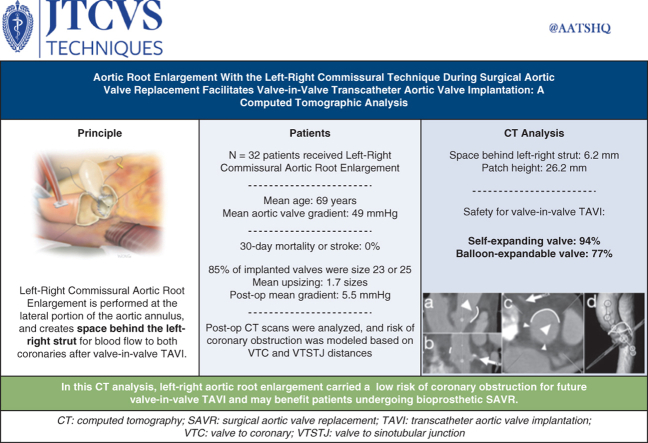

**Figure:**
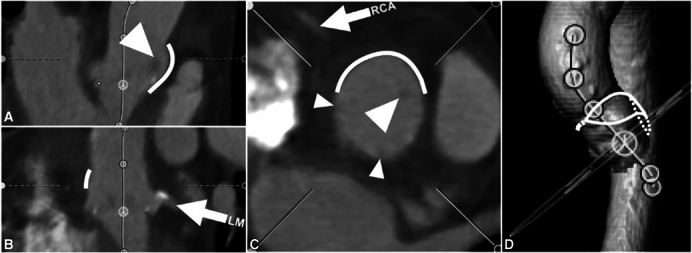
L-R aortic root enlargement mitigates risk of coronary obstruction for future VinV TAVI.

**Central Message**

Left-right aortic root enlargement carries low risk of coronary obstruction for future VinV TAVI and may benefit patients undergoing bioprosthetic SAVR.

**Perspective**

Aortic root enlargement during bioprosthetic aortic valve replacement may reduce likelihood of patient-prosthesis mismatch; however, contemporary posterior techniques may paradoxically hinder future VinV TAVI due to prosthetic tilting toward the coronary arteries. In this CT analysis, we show that the left-right aortic root enlargement mitigates risk of coronary obstruction for future VinV TAVI.

Aortic root enlargement (ARE) mitigates the risk of prosthesis-patient mismatch during surgical aortic valve replacement (SAVR) and is typically performed in the posterior aorta at the noncoronary sinus, as per the Nicks,^1^ Manouguian,^2^ and Yang^3^ methods. ARE may facilitate future valve-in-valve transcatheter aortic valve implantation (VinV TAVI) by creating a larger orifice thus reducing gradients in the new valve.

There is recognition among surgeons and implanters that after ARE the surgical valve sometimes may be slightly canted, usually tilting away from the patch (in the noncoronary sinus) toward the left and right sinuses. Under these circumstances, ARE vexingly may have hindered future VinV TAVI by increasing risk of sinus sequestration and coronary obstruction, an important concern in an anatomically small root with short, effaced sinuses.

A novel technique for ARE, performed on the opposite (lateral) side of the aorta at the left-right commissure (LR),^4^ may mitigate risk of coronary obstruction by creating space behind the LR strut where the patch bulges out, and also in part by permitting the surgical bioprosthesis to cant slightly in the opposite direction, toward the noncoronary sinus (Figure 1).

**Figure 1:**
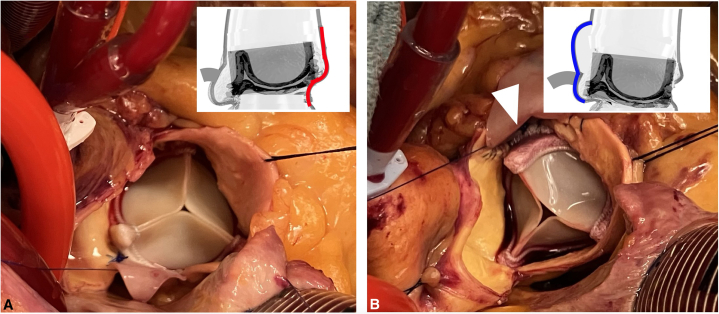
Intraoperative appearance of a posterior (A) and left-right (LR, B) aortic root enlargement (ARE). The surgical valve is often canted away from the patch, toward the right and/or left sinuses in posterior ARE (A), and toward the noncoronary sinus in LR ARE (B). An *arrowhead* indicates space behind the LR valve strut, where the aorta was divided (B). The inset images show stylized aortic root cross-sections (modeled after representative reconstructed CT imaging) with the patch marked in *red* (posterior ARE) and *blue* (LR ARE), and the right coronary artery (RCA) in *solid gray*. When pressurized, the oversized LR patch bulges outwards where the native LR commissure is natively straight co-axially (not shown), as well as over the right sinus (B). The *shaded rectangles (inset*) indicate the cylinder of leaflet tissue (neoskirt) formed during future VinV TAVI; after LR ARE, there is adequate space behind the LR valve strut to permit flow around the neoskirt into both of the adjacent left and right sinuses (B), whereas after posterior ARE the neoskirt may be close to the RCA and/or overlying sinotubular junction. Note the struts on either side of the right sinus (A) abut the aorta, which risks sequestration, despite adequate flow into adjacent sinuses. *CT*, Computed tomography; *VinV TAVI*, Valve-in-valve transcatheter aortic valve implantation.

We planned to analyze the postoperative root anatomy by computed tomography (CT) in a series of patients undergoing LR ARE and to predict whether future VinV TAVI could be safely performed without risk of coronary obstruction and the need for leaflet modification.

## Methods

The Research Ethics Board of Royal Columbian Hospital approved the study protocol and publication of data (no. 2023152, September 7, 2023). Patient written consent for the publication of the study data was waived by the Research Ethics Board, because this was a low-risk retrospective review. Between February 2023 and November 2024, all patients were identified who underwent LR ARE and bioprosthetic SAVR, including other concomitant procedures, by a single surgeon (D.R.W.).

### Operative Technique

**Figure 2:**
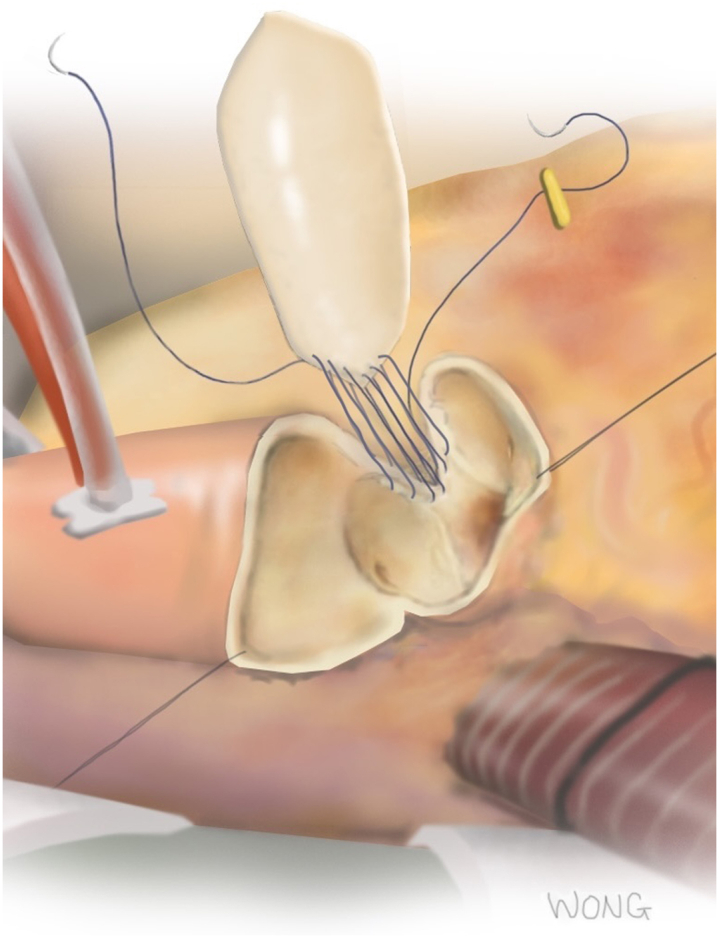
Surgical technique for the left-right (LR) aortic root enlargement (ARE) procedure. The aortotomy is directed toward and through the LR commissure, and a pericardial patch is sewn into the “V”-shaped cleft. It is important to oversize the patch along its width, so there is excess patch material relative to the aortic cleft, permitting the patch to bulge outwards when pressurized.

The technique for LR ARE is described elsewhere.^4^ In brief, the lateral end of the aortotomy is directed toward the LR commissure and down through the subcommissural triangle (Figure 2), which is divided down its midline without entering the muscular septum. The aorta is dissected free from the pulmonary artery, with no need to divide the ligament. An oversized bovine pericardial patch (typically at least 6 × 2.5 cm, beveled on both ends) is sewn into the lateral half of the aortotomy; this is similar to the Nicks method, but on the opposite side of the aorta. The patch, when pressurized, billows outwards to create space behind the LR strut of the bioprosthesis, thus allowing blood flow to enter the left and right sinuses. Patients who needed even more upsizing than is achievable with LR ARE alone were treated with a second simultaneous ARE using the Y technique. A few patients who were already natively sized to a 25-mm valve underwent “partial” LR ARE but without upsizing the valve (that is, the LR patch was sewn in to just above the level of the valve sutures and terminating in the usual area in the ascending aorta). This was done if the surgeon noticed that fully opening the leaflets of the seated surgical valve with an instrument would cause them to contact (or nearly contact) the sinotubular junction (STJ) at either the left or right sinuses. In 7 patients early in the series, a small (<1 × 2 cm) remnant of bovine pericardium was incorporated into the closure of the aortotomy over the left sinus at the STJ, but on CT it did not appear this changed the contour of the aorta here; other than occasional reinforcing pledgeted sutures for needle-hole bleeding there were no other modifications to the described LR ARE technique which might have affected the shape or contour of the patch or aorta, and pledgets were not routinely used.

### Outcomes

Baseline demographics, comorbid conditions, operative details, and 30-day outcomes were tabulated from medical records. Available routine postoperative CT scans were analyzed with 3mensio software (Pie Medical Imaging) to measure the root, including the distances from the virtual valve to both of the coronary ostia (virtual transcatheter valve to coronary, VTC, distance) and to the STJ (virtual transcatheter valve to STJ, VTSTJ, distance) for the left and right sinuses above each ostium. Measurements were modelled for both a self-expanding valve (SEV) and balloon expandable valve (BEV) at time of VinV TAVI, sized per manufacturers' recommendations. For VTC, the virtual transcatheter valve at the level of the coronary was assumed to be constrained by the true internal diameter (ID) of the surgical valve, and without use of balloon valve fracture; for Inspiris Resilia (Edwards Lifesciences) valves, 25 mm or smaller in size with V-Fit technology, the true ID was increased by 2 mm for BEV, but not for SEV (assuming no high pressure pre- or postdilation expanding the ring). The VTSTJ was measured from the virtual transcatheter valve (using nominal diameter for BEV and using the manufacturer's listed diameter at the waist for SEV), and no further outflow flaring of the surgical valve struts was assumed (as might be caused by dilation with larger balloons or volumes, or by dog-boning). The distance from the tip of the LR valve strut to the ARE patch behind it was measured radially; similar measurements were made for the other two struts to the aorta behind each one. The maximum height of the ARE patch and minimum heights of the coronary ostia were measured along the centre line axis relative to the sewing ring. Variation in coronary artery anatomy and the contour/effacement of the sinuses were accounted for whenever they influenced flow to the coronaries.

A VTC of less than 4 mm was considered high risk of coronary obstruction. VTSTJ of less than 2 mm, a risk factor for sinus sequestration, was generally not relevant in scenarios where flow could enter the respective sinuses from behind the LR strut (with at least 2 mm of space). Using these measurements, as well as by qualitatively confirming a route for unobstructed blood flow from the ascending aorta to the coronary, an assessment of high or low risk for coronary obstruction was made to predict safe VinV TAVI. No assumptions were made about future leaflet thickening, tearing, or embolization, thrombus, asymmetric valve expansion or other unpredictable modes of coronary obstruction.

## Results

### Patients

During the study period, 32 patients underwent LR ARE. The majority of patients (62%) were female, and 44% were inpatients requiring urgent/emergency/salvage surgery. All patients had severe, symptomatic aortic stenosis, including 9 bicuspid and one quadricuspid valves, and 4 with moderate or greater regurgitation. There were 2 patients who had a previous 23-mm SAVR requiring redo (including an acutely thrombosed mechanical valve); 1 patient required explant of a 23-mm BEV TAVI due to endocarditis and 1 patient had native valve endocarditis. Average New York Heart Association functional class was 2.7 ± 1.0, and 8 inpatients presented with class 4 symptoms. Additional concomitant procedures included coronary artery bypass (10 patients, 25 total grafts), left atrial appendage exclusion/pulmonary vein isolation (7), myectomy (3), triple valve (with mitral and tricuspid) surgery (2), and redo sternotomy (3) including one third-time sternotomy. Coronary artery disease was present in 11 patients, including 3 with previous percutaneous coronary interventions. Other comorbid conditions are listed in Table 1. One patient with severe claustrophobia could not have CT scans; 1 refused blood products.

**Table 1:** Baseline characteristics and operative details for 32 patients undergoing LR ARE

Age, y, mean ± SD	68.8 ± 9.4
Female	20 (62%)
Body surface area, m^2^, mean ± SD	1.9 ± 0.3
Aortic valve mean gradient, mm Hg, mean ± SD	48.5 ± 13.8
Ejection fraction, %, mean ± SD	56.5 + 8.8
Diabetes mellitus	10 (31%)
Hypertension	20 (62%)
Congestive heart failure	8 (25%)
Coronary artery disease	11 (34%)
Previous myocardial infarction	2 (6%)
Renal failure	2 (6%)
Dialysis	1 (3%)
Chronic pulmonary disease	4 (12%)
Previous stroke	2 (6%)
Previous cardiac surgery	3 (9%)
Cardiopulmonary bypass time, min, mean ± SD	88.0 ± 34.5
Crossclamp time, min, mean ± SD	71.4 ± 31.0
Labeled valve size, mm, mean ± SD	24.3 ± 1.5
21	1 (3%)
23	13 (41%)
25	14 (44%)
27	4 (12%)

Unless otherwise stated, values are reported as n (%). *SD*, Standard deviation.

The majority of patients (27/32, 74%) had ARE with upsizing of the valve, including 7 patients (22%) with concomitant Yang (Y) technique ARE; and the remaining 5 patients (all sized to 25-mm valves before ARE) had a LR ARE patch sewn in above the level of the sewing ring without upsizing the valve but due to risk of sinus sequestration identified intraoperatively. Aortic valves used were Medtronic Avalus valve (n = 1), and Edwards Lifesciences Perimount (n = 5), Magna Ease (n = 7), and Inspiris Resilia (n = 19) valves. No porcine valves were used. Most valves were 23 mm (n = 13) or 25 mm (n = 14).

Before ARE, the annulus measured 21.6 ± 2.7 mm. For the 27 patients with valve upsizing, the annulus measured 20.9 ± 2.4 mm; and the implanted valves represented increases of 1 to 3 valve sizes (1.4 ± 0.5 sizes for LR ARE alone, 2.6 ± 0.5 sizes for combined LR plus Y ARE, 1.7 ± 0.7 sizes for all 27 patients).

### Early Outcomes

For patients with SAVR plus LR ARE only (and no other concomitant procedures), the mean bypass and crossclamp times were 61.4 ± 11.1 minutes and 48.5 ± 6.0 minutes, respectively. Intraoperative mean gradient by transesophageal echocardiogram was 5.5 ± 2.4 mm Hg, and postoperative mean gradient by transthoracic echocardiogram was 5.5 ± 1.8 mm Hg. No patient had greater than trivial postoperative aortic regurgitation. There was no 30-day mortality, stroke, pacemaker implant, or mediastinitis. One patient (3%) needed postoperative dialysis (the patient was on dialysis preoperatively), and 1 patient (3%) had re-exploration for bleeding (not from the aortotomy, patch, or other cardiac sites).

### Computed Tomography (CT) Analysis

**Figure 3:**
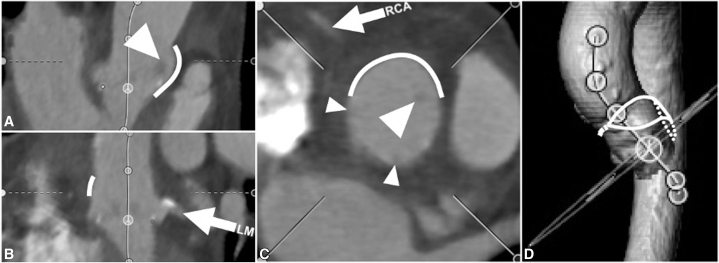
Representative appearance of computed tomography (CT) multiplanar reconstruction of the aortic root after left-right (LR) aortic root enlargement. Orthogonal planar views (A, B, C) show the left (LM) and right (RCA) coronary arteries (*arrows*), the LR valve strut (*large arrowheads*) well away from the patch (*thick line*) behind it, and the LN and RN struts adjacent to the aortic wall (*small arrowheads*). Blood flow entering the right sinus from above, where the patch has expanded the STJ (*thick line* in C), as well as blood flow passing behind the LR strut (between the *large arrowhead* and *thick line* in A and C) into the left sinus, appear to mitigate risk of sinus sequestration during future VinV TAVI. The 3-dimensional CT aortic reconstruction (D) shows the medial portion of the aortotomy (closed primarily) and the location of the patch (*solid* and *dotted outlines*) sewn into the middle and lateral portion of the curved aortotomy, spiraling down through the LR commissure. *VinV TAVI*, Valve-in-valve transcatheter aortic valve implantation.

Postoperative CT scans were available for 31 patients. Root measurements are listed in Table 2. The patch height was 26.2 ± 4.7 mm from the valve sewing ring, with only 1 case (3%) measuring less than 20 mm. The distance behind the LR strut was 6.2 ± 2.4 mm to the patch; visually, this space was continuous with both the L and R sinuses and the ascending aorta above it. The shape of the pressurized patch usually created a slight outward bulge in the contour of the aorta, beginning behind the LR strut and extending above the STJ and over the R sinus (Figure 3). In most cases, the valve and the LR strut appeared to be canted slightly away from the patch. In 1 case where the valve was not appreciably canted, the distance behind the LR strut was <2 mm, but the other struts were >2 mm away from the aorta, permitting flow around the valve in other directions; in no other cases was the distance behind the LR strut <3 mm.

**Table 2:** CT measurements of the aortic root after LR ARE (n = 31)

Root diameter at midsinus		
N to LR	32.1 ± 4.3	
L to RN	32.3 ± 3.8	
R to LN	32.7 ± 3.9	
STJ diameter		
Major axis through patch	34.3 ± 5.8	
Minor axis (perpendicular)	30.0 ± 3.9	
STJ height	15.8 ± 4.2	
Patch height, maximum	26.2 ± 4.7	
VTSTJ distance	L	R
SEV	5.1 ± 2.5	6.5 ± 3.7
BEV	3.7 ± 2.2	5.1 ± 3.7
VTSTJ distance <2 mm, n (%)	L	R
SEV	5 (16%)	2 (6%)
BEV	8 (25%)	5 (16%)
VTC distance	L	R
SEV	7.2 ± 2.2	6.8 ± 2.3
BEV	6.7 ± 2.4	6.3 ± 2.3
VTC distance <4 mm, n (%)	L	R
SEV	1 (3%)	1 (3%)
BEV	4 (13%)	5 (16%)
Distance from tip of surgical strut to aorta		
LR	6.2 ± 2.4	
LN	3.6 ± 2.8	
RN	2.5 ± 2.2	
Coronary height	L	R
	4.7 ± 3.6	5.8 ± 3.6

Unless otherwise stated, values are given in mm, mean ± SD. *N*, Noncoronary cusp; *LR*, left-right commissure; *L*, left cusp; *RN*, right noncommissure; *R*, right cusp; *LN*, left noncommissure; *STJ*, sinotubular junction; *VTSTJ*, virtual transcatheter valve to sinotubular junction; *SEV*, self-expanding valve; *BEV*, balloon-expandable valve; *VTC*, virtual transcatheter valve to coronary.

VTSTJ distances for either coronary measured <2 mm in 6 patients (19%) in the SEV scenario, and in 11 (35%) in the BEV scenario. VTC distances for either coronary measured <4 mm in 2 patients (6%) in the SEV scenario (assumed without balloon inflation); in the BEV scenario, VTC was <4 mm in 7 patients (23%). Of the 11 Inspiris valves (size ≤25 mm) implanted in patients with valve upsizing, L and R VTC measurements would decrease from 6.4 ± 1.6 mm and 6.0 ± 1.8 mm to 5.4 ± 1.6 mm and 5.0 ± 1.8 mm, respectively, after assumed VFit ring expansion with ballooning; 5 of these patients would have at least 1 VTC distance of <4 mm after balloon expansion, whereas none would have had VTC <4 mm in the unexpanded state. All 5 patients who underwent “partial” LR ARE without valve upsizing, had VTC >4 mm with either BEV or SEV, and 2 of 5 had VTSTJ measurements between 2-3 mm with BEV.

On the basis of these measurements, 94% of patients would be expected to have a safe VinV TAVI with a SEV (without significant risk of coronary obstruction). If balloon inflation were performed with a SEV, or if a BEV were used, 77% could have safe VinV TAVI, due to assumed expansion of Inspiris Resilia valves (59% of patients in this series) reducing VTC.

## Discussion

There has been a steady temporal increase in the use of bioprosthetic versus mechanical valves, including among younger patients who may survive beyond the expected valve durability.^5^ Furthermore, rates of ARE usage during SAVR are increasing,^6^ as are rates of VinV TAVI to treat failing bioprostheses.^7^ Taken together, these trends suggest that understanding the implications of ARE on the feasibility of VinV TAVI is essential. ARE techniques, like the LR ARE method, intended to mitigate risk of future coronary obstruction need to be assessed rigorously with CT imaging to determine whether they achieve the desired effects and facilitate safe VinV TAVI.

In this study, the LR ARE technique indeed would permit future VinV TAVI, with up to a 94% likelihood of avoiding coronary obstruction, by all but eliminating the risk of sinus sequestration at the STJ level, even in a patient population with natively small roots. Adequate room for blood flow behind the LR strut was achieved. Coronary obstruction (based on VTC <4 mm) would still occur in 2 (6%) patients, although this rate is higher (23%) if balloon inflation (as with BEV or pre- and postdilation) is used and results in expansion or fracture of the surgical valve, including expanding Inspiris Resilia valves. Patent bypass grafts may mitigate this risk in some patients (in our series the risk of low VTC for BEV would decrease from 23% to 17%). The 2 cases with inadequate VTC occurred within the first 5 patients during the steepest part of our learning curve, and the risk of coronary obstruction was 0% for the next 27 patients. Although the likelihood of coronary obstruction is estimated to be 2% to 6% among VinV TAVI all-comers,^8^^,^^9^ the population of patients requiring ARE is likely to be at greater baseline risk of coronary obstruction. From these data, VTSTJ distances were inadequate (<2 mm) in 25% in this series, and were it not for the space between the LR strut and the patch, this would have led to sinus sequestration in at least a quarter of patients otherwise. It may be difficult to compare these result to those with other techniques. Nevertheless, in one published series, 36% of patients after a Y technique ARE had inadequate VTC <4 mm at the right coronary artery, and 9% were inadequate at the left main, but the combined risk for either coronary ostium was not quoted in the manuscript.^10^

We provide a high-fidelity quantitative analysis of the aortic root assessing feasibility of VinV TAVI, exploring both SEV and BEV options, taking into account constraint at the level of surgical sewing ring on VTC^11^ and full expansion at the tips of the struts,^12^ and measuring the distance behind the LR strut. Whereas small VTSTJ dimensions are usually indicative of a high risk of sinus sequestration, after LR ARE the tall height of the patch combined with robust space behind the LR surgical valve strut are more relevant to providing adequate flow to the sinuses, especially for the left sinus where the VTSTJ is not as large. Because of the 3-dimensional curvature of the aortotomy and aorta, the patch tends to allow the top of the right sinus to bulge outwards too, usually resulting in generous VTSTJ on the right, which also makes catheter access to the right coronary artery easier. In some cases, the left ostium may be challenging to access for catheter interventions due to small VTSTJ distances.

Neoskirt height (with leaflets in the fully open position) for a 26-mm Evolut SEV is 26.7 mm tall; even for a tall frame valve, assuming a usual implant depth of 4 to 6 mm for VinV TAVI, the average height of the patch (26.2 mm) is high enough that there should be adequate flow over the tall frame valve neoskirt into the space behind the LR strut. Thus, TAVI-in-TAVI-in SAVR should also theoretically be possible for the vast majority of patients (only 3% of patients had a maximum patch height of less than 20 mm), with the same caveats regarding the effect of expanding/fracturing the surgical valve on VTC applicable to both TAVI procedures.

The LR ARE technique can be used in various ways: (1) as a stand-alone ARE procedure to upsize the valve 1 to 2 sizes; (2) in combination with posterior ARE procedures, whenever more upsizing is needed, especially to counter potential canting toward the L and R sinuses; and, (3) as a “partial” ARE to modify the root for future VinV TAVI but without any valve upsizing (this can even be performed after the valve has been seated, but is easiest done if the aortotomy is already directed to the top of the LR commissure). Unlike posterior root enlargements, LR ARE is not influenced by the presence of mitral prostheses and does not risk inducing mitral regurgitation; it is also relatively quick to perform with superb exposure. Short-term outcomes demonstrated acceptably low risk of complications. Because closing the aortotomy is largely equivalent to sewing one edge of the patch, not much additional suturing is required to perform ARE. Aiming the aortotomy toward the LR commissure is easy to do and ideally surgeons could consider doing this for every SAVR case. Because the principle of creating additional space around the valve in a vital location adjacent to the L and R sinuses is so important, there is a rational argument for routinely adding a “partial” or full LR ARE in almost all SAVR cases, unless the root is already generous and ectatic.

### Limitations

This study is limited by being a single-surgeon series with strictly bovine pericardial prostheses, and incorporating a combination of enlargement techniques. Other good options such as root replacement were not included in this series. No controls without ARE were available for comparison. Although we considered the implications of both BEV and SEV in this analysis, balloon valve fracturing was not modeled as the degree of enlargement in the true ID is quite unpredictable; nor did we account for potential scenarios of obstruction like torn or embolized leaflet tissue or thrombus, interactions of tall frame BEV or the inflation balloon of SEV with the curvature and size of the ascending aorta causing canting of the TAVI valve within the surgical valve, or the effect of various (higher/lower) balloon inflation volumes, constraint of the balloon causing a high degree of dog-boning, implantation depths, and strut compliance on outflow flaring greater than the full nominal size of BEV or on expansion of Inspiris Resilia valves beyond an estimated 2 mm expansion. The available postoperative CT studies were not performed using high-resolution, thin-slice protocols typically used for TAVI planning, which likely would have improved precision and reduced some artifact. This series was the earliest experience with the LR ARE technique, and further evolution in the technique and improved knowledge may lead to further refinement. Finally, it is possible that a cut-off value VTC of <4 mm may be overly conservative, as it was derived without including VTSTJ in the multivariable model,^8^ and indeed a number of the controls (without obstruction) in that paper had VTC values < 4 mm; as a result, this cut-off may have been confounded by insufficient VTSTJ causing sinus sequestration, which is all but a moot issue after LR ARE, and perhaps a lower cut-off may better predict obstruction risk once VTSTJ is accounted for (perhaps using <2 mm like is used for VTSTJ). Therefore, it is possible that more than 94% of our cases might possibly be safe for future VinV TAVI. Conversely, other factors (above) may reduce this proportion below 94%.

## Conclusions

The LR ARE technique appeared to facilitate future VinV TAVI in the great majority of patients by reducing risk of sinus sequestration. This technique may be a useful adjunct to SAVR in patients with small root anatomy and may have benefits more broadly to facilitate future VinV TAVI in the general SAVR population.

## Conflict of Interest Statement

D. Wong has received consulting and/or proctoring fees from Edwards Lifesciences, Artivion, and Boston Scientific. All other authors reported no conflicts of interest.

The *Journal* policy requires editors and reviewers to disclose conflicts of interest and to decline handling or reviewing manuscripts for which they may have a conflict of interest. The editors and reviewers of this article have no conflicts of interest.
